# Clinical and epidemiological features of pertussis in Salvador, Brazil, 2011–2016

**DOI:** 10.1371/journal.pone.0238932

**Published:** 2020-09-11

**Authors:** Lucas Oliveira Araújo, Amélia Maria Pithon Borges Nunes, Viviane Matos Ferreira, Cristiane Wanderley Cardoso, Caroline Alves Feitosa, Mitermayer Galvão Reis, Leila Carvalho Campos

**Affiliations:** 1 Instituto Gonçalo Moniz, FIOCRUZ, Salvador, Bahia, Brazil; 2 Escola Bahiana de Medicina e Saúde, Salvador, Bahia, Brazil; 3 Secretaria Municipal de Saúde, Salvador, Bahia, Brazil; 4 Faculdade de Medicina, Universidade Federal da Bahia, Salvador, Bahia, Brazil; 5 Department of Epidemiology of Microbial Diseases, Yale School of Public Health, New Haven, Connecticut, United States of America; University of Georgia, UNITED STATES

## Abstract

Pertussis, a severe respiratory infection caused by *Bordetella pertussis*, is distributed globally. Vaccination has been crucial to annual reductions in the number of cases. However, disease reemergence has occurred over the last decade in several countries, including Brazil. Here we describe the clinical and epidemiological aspects of suspected pertussis cases in Salvador, Brazil, and evaluate factors associated with case confirmation. This descriptive and retrospective study was conducted in the five hospitals in Salvador that reported the highest number of pertussis cases between 2011–2016. Demographic and clinical data were recorded for each patient. Bivariate analysis was performed to evaluate differences between groups (confirmed vs. unconfirmed cases) using Pearson’s Chi-square test or Fisher’s exact test. Results: Of 529 suspected pertussis cases, 29.7% (157/529) were confirmed by clinical, clinical-epidemiological or laboratory criteria, with clinical criteria most frequently applied (63.7%; 100/157). Unvaccinated individuals (43.3%; 68/157) were the most affected, followed by age groups 2–3 months (37.6%; 59/157) and <2 months (31.2%; 49/157). Overall, ≤50% of the confirmed cases presented a complete vaccination schedule. All investigated cases presented cough in association with one or more symptoms, especially paroxysmal cough (66.9%; 105/529) (p = 0.001) or cyanosis (66.2%; 104/529) (p<0.001). Our results indicate that pertussis occurred mainly in infants and unvaccinated individuals in Salvador, Brazil. The predominance of clinical criteria used to confirm suspected cases highlights the need for improvement in the laboratory tools used to perform rapid diagnosis. Fluctuations in infection prevalence demonstrate the importance of vaccination strategies in improving the control and prevention of pertussis.

## Introduction

Pertussis is an acute respiratory disease mainly caused by the Gram-negative bacterium *Bordetella pertussis*. This illness is characterized by a prolonged paroxysmal cough, which can be frequently associated with vomiting, fever, cyanosis and apnea [1]. While disease presentation can vary with age, history of previous exposure and vaccination, the incidence of pertussis in young infants is of particular concern, as infants are at the highest risk of death and developing severe disease [2, 3].

Although the introduction of vaccines has significantly reduced the pertussis burden, epidemic waves have continued to occur in cycles ranging from every 2 to 5 years [2, 4]. In 2018, despite an estimated 86% global inactivated whole cell (wP) or acellular (aP) vaccine coverage administered through three primary doses, the World Health Organization (WHO) reported 151,074 cases of pertussis [5]. Today, pertussis continued to represent a serious public health problem in many countries, even in those with high rates of vaccination coverage [6].

The Brazilian Ministry of Health, through the National Immunization Program (PNI-MS), recommends the administration of the diphtheria, tetanus, whole cell pertussis (DTwP) + *Haemophilus influenzae* type b (Hib) + Hepatitis B (Hb) pentavalent vaccine for infants aged two, four and six months. Subsequently, two boosters of the diphtheria-tetanus-whole pertussis vaccine (DTwP) are recommended for children at 15 months of age and between the ages of 4–6 years. Pregnant women are generally advised to receive a booster with reduced antigen content of diphtheria, tetanus and acellular pertussis vaccine (Tdap) between the 20th and 36th weeks of gestation [7–9].

In Brazil, the notification of pertussis is mandatory. All suspected cases must be reported and investigated, and data on these published in the National Information System for Notifiable Diseases (SINAN) of the Brazilian Ministry of Health [10]. Suspected pertussis cases are confirmed based on the following criteria: 1) isolation of *B*. *pertussis* in cultures of nasopharyngeal secretion and/or the detection of bacterial DNA by real-time (quantitative) polymerase chain reaction (qPCR) (laboratory criteria); 2) reported close contact with a laboratory-confirmed case (clinical-epidemiological criteria); 3) the presentation of two or more suggestive symptoms (e.g. paroxysmal cough, cyanosis, stridor, vomiting after coughing, apnea) in association with complementary examinations (e.g. lymphocytosis and leukocytosis) (clinical criteria) [11, 12].

From 2007 to 2014, a total of 80,068 suspected and 24,612 confirmed cases of pertussis were reported in Brazil, with a significant increase in incidence observed between 2012–2014 [10–12]. A peak was observed in 2014 (4.03/100,000 inhabitants), with the highest incidence noted children in their first year of life [13, 14].

In Salvador, Brazil, according to the Health Secretariat of the State of Bahia (SESAB), 72 health institutions reported 1,101 suspected cases of pertussis between 2011–2016. From 2011 to 2014, there was an increase in the number of suspected cases, from 64 in 2011 to 494 in 2014, followed by a decline to 158 and 56 cases in 2015 and 2016, respectively [14, 15]. In 2015, over 95% coverage of the pentavalent and DTwP vaccines was achieved, which subsequently declined to 87.31% (pentavalent) and 91.67% (DTwP) in 2016 [16]. In 2014, the tetanus-reduced diphtheria toxoid-acellular pertussis (Tdap) vaccine was introduced in Brazil, which continues to be recommended for pregnant women, health-care professionals and children who develop serious side effects after receiving the wP vaccine [7–9]. In 2015 and 2016, Tdap vaccine coverage remained below 60% among pregnant women [16].

The analysis of reported cases of a disease, such as pertussis, can contribute to an enhanced understanding of the underlying epidemiology, and to improvements in control and prevention measures. This study aimed to describe the clinical and epidemiological aspects of suspected pertussis cases reported between 2011 and 2016 by five of the main hospitals in Salvador, Bahia, as well as to analyze the factors associated with the confirmation of these cases.

## Materials and methods

### Ethical approval

The study was approved by the Ethics Committee at the Gonçalo Moniz Institute, FIOCRUZ-BA (CAEE # 54392116.3.0000.0040) and was conducted in accordance with good clinical practices.

### Study design and population

This descriptive and retrospective study was conducted in Salvador, the capital of the state of Bahia (estimated population of 2.9 million in 2016, fourth city of Brazil), located in the northeastern region of Brazil. All suspected cases of pertussis reported by five hospitals in Salvador were investigated during the period of 2011–2016 (S1 Database). These hospitals were selected due to high numbers (≥75 cases/hospital) of suspected pertussis cases during the study period (representative total: 50.9%; 560/1,101 cases reported in the city of Salvador). This study included periods three years before and two years after dTap recommendation in the maternal immunization by PNI in 2014 [8].

### Data collection

Data was obtained from the National Information System for Notifiable Diseases (SINAN), and a database maintained by the Epidemiological Surveillance Department (DIVEP) of SESAB.

The selected hospitals were contacted and requested to provide the medical records of all suspected pertussis cases when available. Suspected cases were reported in accordance with clinical and epidemiological criteria, following the Brazilian Ministry of Health guidelines [12].

Sociodemographic and epidemiological data were collected using a comprehensive standardized form (S1 Research form), including age, sex, vaccination status, prevention and control measures (e.g. chemoprophylaxis and vaccination are recommended for contacts of suspected pertussis cases) and other clinical data (e.g. symptoms and complications). To ensure accuracy, double data entry was performed, followed by validation and management using the REDCap (Research Electronic Data Capture) electronic data capture tool [17].

### Statistical analysis

Bivariate analysis was performed to evaluate differences between clinical characteristics in confirmed vs unconfirmed groups, in addition to clinical symptoms/complications and sociodemographic variables (e.g. age, sex and vaccination status). The age variable was divided into six categories, based on eligibility according to the pertussis vaccine schedule [14]: <2 months; ≥ 2 and <4 months; ≥ 4 and <6 months; ≥ 6 months and <1 year; ≥ 1 and <4 years and ≥ 4 years. Comparisons between groups (confirmed vs unconfirmed) were performed using Pearson’s Chi-squared test or Fisher’s Exact test. Statistical significance was considered when p <0.05. All data analyses were conducted using STATA v12 software (College Station, Texas).

## Results

Of the 560 cases selected, 529 (94.5%) were included, while 31 (5.5%) were excluded due to a complete absence of medical records. Of these, 52% (275/529) of the patients were aged less than four months (Table 1). For one of the hospitals, 14 more cases were identified, in addition to those reported.

**Table 1:** Sociodemographic and epidemiological characteristics of 529 suspected pertussis cases in Salvador, Brazil (2011–2016).

Characteristics	Pertussis suspected cases	
	Total number	%
**Age group**		
< 2 months	115	21.7
≥ 2 months < 4 months	160	30.3
≥ 4 months < 6 months	83	15.7
≥ 6 months < 1 year	60	11.3
≥ 1 year < 4 years	38	7.2
≥ 4 years	73	13.8
**Sex**		
Male	243	45.9
Female	286	54.1
**Collected nasopharyngeal sample for culture**		
Yes	407	76.9
No	117	22.1
No information	5	1.0
**Antibiotic prescribed?**		
Yes	461	87.2
No	60	11.3
No information	8	1.5
**Contact with suspected/confirmed pertussis case**		
Yes	41	7.8
No	185	35.0
No information	303	57.2
**Control and prevention measures** ^a^		
Yes	44	8.3
No	400	75.6
No information	85	16.1

^a^ Chemoprophylaxis and vaccination are recommended for contacts of suspected pertussis cases.

Suspected pertussis cases were almost similarly distributed according to sex, with a light prevalence in females (54.1%; 286/529). In 407 cases, nasopharynx samples were collected for culture (76.9%; 407/529). Antibiotics were prescribed in 87.2% of suspected cases (461/529). Only 7.8% (41/226) individuals were reported as contact with another suspected or confirmed pertussis case. Control and prevention measures were performed for 8.3% (44/529) patient contacts (e.g. family members, intimate contacts) (Table 1).

**Fig 1:**
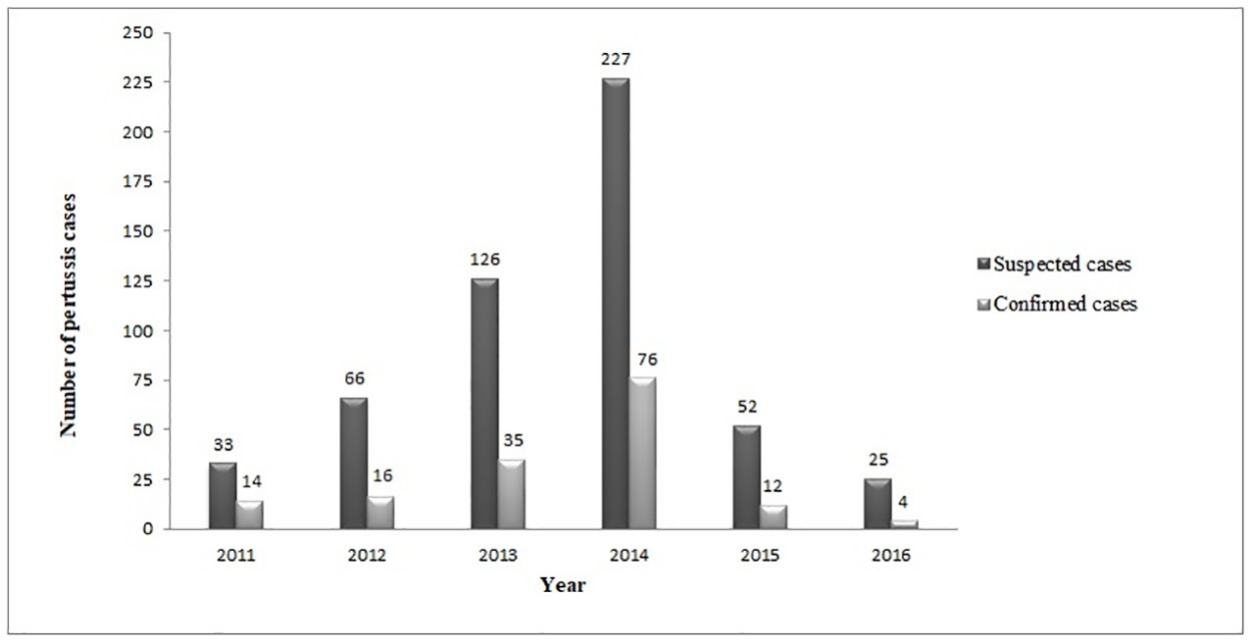
Number of suspected and confirmed pertussis cases in Salvador, Brazil (2011–2016). Suspected cases: individuals who presented cough in association with one or more symptoms of disease; Confirmed cases: pertussis cases considered as confirmed according to Brazilian Ministry of Health guidelines [12].

A 6.9-fold increase in the number of pertussis cases was reported between 2011–2014, from 33 (6.2%; 33/529) suspected cases in 2011 to a peak of 227 cases (42.9%; 227/529) in 2014 (Fig 1).

From 2015–2016, the number of reported pertussis cases successively declined, with 52 (9.8%; 52/529) and 25 (4.7%) suspected cases in each year, respectively. A similar pattern was observed in numbers of confirmed pertussis cases (Fig 1).

A total of 29.7% (157/529) of the reported cases were confirmed by clinical, clinical-epidemiological or laboratory criteria. Most suspected cases were confirmed by clinical criteria (63.7%; 100/157), followed by 26.7% (42/157) and 9.6% (15/157) by laboratory and clinical-epidemiological criteria, respectively. A total of 206/372 (55.4%) pertussis cases were discarded due to laboratory analysis (Table 2).

**Table 2:** Characteristics of confirmed and unconfirmed pertussis cases in Salvador, Brazil (2011–2016) (N = 529).

Characteristics	Number (%) of confirmed cases	Number (%) of unconfirmed cases	*p-value*
	(*n* = 157)	(*n* = 372)	
**Age group**			
< 2 months	49 (31.2)	66 (17.7)	<0.001^a^
≥ 2 months < 4 months	59 (37.6)	101 (27.2)	
≥ 4 months < 6 months	17 (10.8)	66 (17.7)	
≥ 6 months < 1 year	17 (10.8)	43 (11.6)	
≥ 1 year < 4 years	4 (2.6)	34 (9.1)	
≥ 4 years	11 (7.0)	62 (16.7)	
**Sex**			
Male	78 (49.7)	165 (44.4)	0.261^b^
Female	79 (50.3)	207 (55.6)	
**Confirmation criteria**			
Laboratory	42 (26.7)	206 (55.4)	<0.001^b^
Clinical-epidemiological	15 (9.6)	29 (7.8)	
Clinical	100 (63.7)	137 (36.8)	
Symptom*			
Cough	157 (100.0)	372 (100.0)	-
Paroxysmal Cough	105 (66.9)	189 (50.8)	0.001^b^
Stridor	42 (26.8)	79 (21.2)	0.168^b^
Fever	40 (25.5)	107 (28.8)	0.441^b^
Cyanosis	104 (66.2)	162 (43.5)	<0.001^b^
Vomiting after coughing	54 (34.4)	146 (39.2)	0.293^b^
Apnea	17 (10.8)	39 (10.5)	0.906^b^
Dyspnea	44 (28.0)	94 (25.3)	0.509^b^
Other	6 (3.8)	12 (3.2)	0.730^b^
**Presence of complications**			
Yes	17 (10.8)	74 (19.9)	0.012^b^
No	140 (89.2)	298 (80.1)	
Type of complication*			
Pneumonia	7 (41.1)	47 (63.5)	0.005 ^a^
Encephalopathy	0 (0.0)	7 (9.5)	0.084 ^a^
Otitis	1 (5.9)	1 (1.4)	0.506 ^a^
Innutrition	1 (5.9)	8 (10.8)	0.199 ^a^
Dehydration	4 (23.5)	14 (18.9)	0.340 ^a^
Other**	5 (29.4)	7 (9.5)	0.358 ^a^
**Collection of nasopharyngeal sample for culture**			
Yes	106 (67.5)	301 (80.9)	0.002^b^
No	50 (31.8)	67 (18.0)	
No information	1 (0.6)	4 (1.1)	
**Antibiotic prescribed?**			
Yes	145 (92.4)	316 (84.9)	0.063^b^
No	11 (7.0)	49 (13.2)	
No information	1 (0.6)	7 (1.9)	
**Vaccination (complete/partial)**			
Yes	56 (35.7)	158 (42.5)	0.004^b^
No	68 (43.3)	105 (28.2)	
No information	33 (21.0)	109 (29.3)	

^a^Fisher’s exact test

^b^Pearson’s chi-square test

* One case had more than one symptom and complication.

** Anemia, atelectasis, bronchiolitis, hypoxia, hypoxemia, pneumothorax, cardiorespiratory arrest or septicemia.

Of 157 pertussis confirmed cases, 37.9% (59/157) and 31.2% (49/157) of the patients were aged between 2–4 months and <2 months, respectively. In contrast, unconfirmed cases were uniformly distributed among the age groups. No statistically significant differences were observed with respect to sex among the confirmed and unconfirmed cases (Table 2).

All suspected pertussis cases (confirmed and unconfirmed) presented symptomatic cough, while higher percentages of paroxysmal cough (66.9%; 105/157) (p = 0.001) and cyanosis (66.2%; 104/157) (p<0.001) were observed among the confirmed pertussis cases (Table 2). A comparison of confirmed and unconfirmed cases according to laboratory test confirmation revealed that cyanosis (p = 0.006) was the only statistically significant symptom between these groups (data not shown). It is important to note that the low sample number in both groups may have made statistical analysis and interpretation of the results challenging.

Although relatively rare, 10.8% of confirmed pertussis cases presented complications, of which pneumonia (41.1%; 7/17) was the most prevalent (Table 2). Antibiotics were prescribed in a high proportion of confirmed (92.4%; 145/157) and unconfirmed (84.9%; 316/372) pertussis cases, while nasopharynx sample collection was more frequent among unconfirmed pertussis cases (80.9%; 301/372) (Table 2).

Statistically significant differences regarding vaccination status (p = 0.004) were observed between the confirmed and unconfirmed groups. Approximately 36% (56/157) of the confirmed cases had been vaccinated against pertussis, and 28.0% (44/157) had received all doses according to schedule (Table 3). In contrast, 42.5% (158/372) of unconfirmed cases were vaccinated (Table 2). Overall, ≤50% of the confirmed and unconfirmed cases presented a complete vaccination schedule for age (Table 3).

**Table 3:** Distribution of the number of vaccine doses in confirmed and unconfirmed pertussis cases, according to age group (N = 529).

Cases	Age group	n (%)	Number of vaccine doses^a^					Complete vaccination schedule for age (%)
			1D	2D	3D	3D+1B	3D+2B	
Confirmed (*n* = 157)	< 2m	49 (31.2)	N/A	N/A	N/A	N/A	N/A	N/A
	≥ 2m < 4m	59 (37.6)	28	N/A	N/A	N/A	N/A	47.5
	≥ 4m < 6m	17 (10.8)	6	7	N/A	N/A	N/A	41.2
	≥ 6m < 1y	17 (10.8)	1	-	7	N/A	N/A	41.2
	≥ 1y < 4y	4 (2.6)	-	1	1	2	N/A	50.0
	≥ 4y	11 (7.0)	-	-	1	-	-	-
Unconfirmed (*n* = 372)	< 2m	66 (17.7)	N/A	N/A	N/A	N/A	N/A	N/A
	≥ 2m < 4m	101 (27.2)	50	N/A	N/A	N/A	N/A	49.5
	≥ 4m < 6m	66 (17.7)	23	22	N/A	N/A	N/A	33.3
	≥ 6m < 1y	43 (11.6)	2	8	20	N/A	N/A	46.5
	≥ 1y < 4y	34 (9.1)	2	1	11	7	N/A	20.6
	≥ 4y	62 (16.7)	-	-	-	3	10	16.1
	Total	529 (100.0)						

^a^The doses of the following vaccines were considered: triple bacterial cell (DTP—diphtheria, tetanus and pertussis); tetravalent (DTP + Hib—Diphtheria, tetanus, pertussis and *Haemophilus influenzae* type b) and pentavalent (DTP + Hib + Hb—Diphtheria, tetanus, pertussis, *Haemophilus influenzae* type b and Hepatitis B); D = dose; B = booster.

^b^ N/A = not applicable

## Discussion

The present study describes clinical and epidemiological aspects associated with pertussis cases as reported by five hospitals in the city of Salvador between 2011 and 2016. In this period, important aspects were associated with the occurrence of this disease in Brazil, as reflected in Salvador: an increase of pertussis cases was noted in 2012, with high numbers of reported cases, reaching a peak in 2014. After 2014, reductions in the number of reported cases were seen through 2016 [11, 13].

Multiple factors may underlie the increased number of suspected pertussis cases reported during 2011–2014. Firstly, as immunization coverage was low and the expected vaccination coverage of 95% from three DTP (or pentavalent) primary doses was only achieved in 2015, this correlates with the reduction in the number of suspected cases reported between 2015–2016. Second, increased disease surveillance in some states, including Bahia, could have led to increased numbers of suspected case notifications [11, 15]. In addition, the literature contains some hypotheses attempting to explain the resurgence of pertussis in many countries, including increased awareness of the disease, decreased immunity conferred by vaccination and/or natural infection, the switch from wP to aP vaccines, and antigenic variability in *B*. *pertussis* strains [18–20]. Some of the main public health challenges have been evidence of waning immunity in current vaccines, lack of compliance with the vaccine schedule and conducting targeted immunization campaigns to protect susceptible populations [9, 21]. Also, it is important to note that pertussis epidemiology indicates that epidemic cycles normally occur every 2–5 years in Brazil, similar to what is observed in other countries [2, 13].

Despite low immunization coverage in mothers (<60%), the inclusion of the dTap vaccine for pregnant women in 2014 may have contributed to a reduction in the number of pertussis cases during 2015–2016 [13, 16]. It is known that a single dose of dTap during pregnancy enhances antibody levels in the mother, which should provide passive protection to her newborn in the first months of life [22, 23]. Accordingly, several studies have recommended increased vaccination and immunization monitoring in pregnant women; increased awareness campaigns would serve to make this target population more aware of the benefits that vaccination offers to protect their babies [13, 22, 23].

The high percentage of confirmed cases among infants aged four months or less was expected, as these children do not present a sufficient immune response to confer pertussis protection, and are thus not eligible for a complete vaccination schedule [7, 12, 24]. However, similar rates of vaccine coverage between confirmed and unconfirmed cases was surprising. Considering that non-specific symptoms of pertussis constitute a mandatory notification of a suspected case of pertussis, it is possible that this procedure could introduce bias into the classification of pertussis cases. Moreover, several studies have shown a higher frequency of pertussis in children aged one year or less, mainly those under six months, who present high numbers of hospitalizations and more frequent disease complications [1, 13, 24–27].

Similarly to other studies conducted in Brazil [1, 24], we found no difference in disease occurrence with respect to sex. However, it is interesting to note that other investigations have reported a predominance of pertussis cases in females [28, 29] or males [27, 30], despite a lack of statistical significance.

The prevalence of cases confirmed by clinical criteria in this study is consistent with surveillance conducted throughout the country [11], which may introduce bias in the true number of confirmed cases, since this may reflect the influence of relevant factors, including low symptoms in some age groups, antibiotic use and vaccination status [2, 4].

Although laboratory diagnosis is considered the gold standard for pertussis confirmation [12], its use remains limited throughout the country [11]. According to SESAB, the performance of laboratory confirmation of pertussis cases is hindered by difficulties in sample collection at many hospitals and health centers, coupled with the appropriate transport of samples to the Central Laboratory of Public Health (LACEN), located in Salvador, Bahia [31], where laboratory testing is performed. This calls attention to the importance of improving the availability of necessary laboratory tools for performing rapid pertussis diagnostics. In fact, since 2009, Brazil and other Latin American countries, including Argentina, Chile and Mexico, have taken part in the LAPP (“Latin America Pertussis Project”) initiative, which aims to improve the epidemiological surveillance of pertussis by expanding and enhancing laboratory diagnosis procedures [32].

The use of antibiotics was predominant among the confirmed cases, which is consistent with the guidelines established by the Brazilian Ministry of Health [12]. A report in the literature states that early antibiotic therapy may contribute significantly to a reduction in the transmission period and to disease remission [33].

Paroxysmal cough and cyanosis were found to be the most frequent and statistically significant symptoms associated with confirmed cases of pertussis herein. However, stridor was not significant in our results, despite being commonly referred to in the literature [24, 26].

Approximately 10% of the confirmed cases herein presented complications, such as pneumonia. This condition has been associated with whooping cough and a severe clinical status [11, 24, 34]. Fortunately, cure was the predominant outcome and no pertussis deaths were identified among the patients investigated during the study period. Indeed, the prognosis is generally good in most confirmed pertussis cases, with a typical mortality rate of around 2% [11, 27, 35].

We identified a high frequency of confirmed cases among unvaccinated patients, and a low percentage of patients presenting an age-appropriate complete vaccination schedule. Our results highlight the importance of vigilance in vaccination, particularly with regard to susceptible individuals, in order to encourage the completion of the vaccination schedule, which consequently contributes to reductions in the number of cases [4, 36, 37].

This descriptive and retrospective study was subject to some limitations. As the study was conducted in just five local hospitals, the results may not be representative of the overall pertussis disease scenario considering the entire population of the Salvador metropolitan area. Moreover, the review of medical records implies the analysis of secondary data that is not updated, and which may commonly be subject to underreporting. In this regard, it is important to point out that not all medical records were available for analysis; in at least one instance, greater numbers of pertussis cases were identified than were present in the national surveillance system database. Our results are further limited by the absence and/or improper recording of information, which unquestionably affects the quality and reliability of the collected data. For example, we were unable to identify the vaccination status of the mothers with diseased children <1 year of age, and hospitalization information (yes or no) was also occasionally missing. On the other hand, the occurrence of missing data dramatically highlights the need to improve appropriate recordkeeping procedures and accurately notify pertussis cases, and also calls attention to the importance of training health care professionals with regard to these procedures and the use of national and state surveillance systems.

## Conclusions

The present study revealed a similar pattern of pertussis infection as reported elsewhere, which mostly occurs in infants and unvaccinated individuals. The present results highlight the need to improve data recordkeeping and epidemiological surveillance efforts, as well as provoke further discussion surrounding disease confirmation criteria and control strategies. Moreover, we emphasize the importance of vaccination strategies in reducing the burden of severe disease, particularly in susceptible young infants and pregnant women, and reinforce the significance of continuing epidemiological surveillance to better understand the reemerge of pertussis.

## Supporting information

S1 Database Pertussis cases analyzed in Salvador, Brazil (2011–2016). (XLS)

S1 Research form (DOCX)
