# Supplementary material for: Clinical and epidemiological features of pertussis in Salvador, Brazil, 2011–2016
Source: PLoS One. 2020 Sep 11;15(9):e0238932. doi: 10.1371/journal.pone.0238932 (PMC7485779; doi:10.1371/journal.pone.0238932)
Supplement: S1 Research form — (DOCX) [file pone.0238932.s002.docx]

| Sociodemographic data | Participant number: | | | |
| --- | --- | --- | --- | --- |
|  | Age at admission: | Sex  □Female  □Male | Pregnant  □Yes □No | Skin color  □  1-White 2-Black 3-Yellow 4-Mixed-race 5-Indigeneous 9-Ignored |
|  | Municipality of Residence: | | Occupation: | |

| Epidemiological Background | Month and year of diagnosis of suspected case  ___/___ | Contact with a suspected or confirmed pertussis case  (up to 14 days prior to onset of signs and symptoms)  □Yes □No | |
| --- | --- | --- | --- |
|  | Contact Location  □  1-Household 2-Neighborhood 3-Work 4-Day Care/School 5-Health Center/ Hospital 6-Other State/Municipality 7-Other Locations: | | Relationship with Contact  □Mother □Father □Brother/Sister  □ Uncle/Aunt □Grandparents |
|  | Vaccine Type | Number of vaccine doses | |
|  | □DTP | □1-One 2-Two 3-Three 4-Three+Booster 5-Three+Two Boosters 6- Never vaccinated 9-Ignored | |
|  | □DTP+Hib | □1-One 2-Two 3-Three 4-Three+Booster 5-Three+Two Boosters 6- Never vaccinated 9-Ignored | |
|  | □DTP+Hib+HepB | □1-One 2-Two 3-Three 4-Three+Booster 5-Three+Two Boosters 6- Never vaccinated 9-Ignored | |
|  | □DTPa | □1-One 2-Two 3-Three 4-Never vaccinated 9-Ignored | |

| Clinical Data | Month and year of cough onset  ___/___ | | |
| --- | --- | --- | --- |
|  | Signs and Symptoms  □Yes  □No  □Ignored | □Cough  □Paroxysmal Cough  □ High-pitched sound at the end of coughing (Whoop; Stridor)  □Fever | □Cyanosis  □Vomiting  □Apnea  □Others ________ |
|  | Complications  □Yes  □No  □Ignored | □ Pneumonia or Bronchopneumonia  □ Encephalopathy (convulsion)  □ Dehydration  □Otitis  □Malnutrition  □Others ________ □Ignored | |

| Care, Treatment and Laboratory Data | Hospitalization  □Yes □No □Ignored | Month and year of hospitalization  ___/___ | | Hospital  __________________ |
| --- | --- | --- | --- | --- |
|  | Antibiotic Prescribed?  □Yes □No □Ignored | Month and year of antibiotic administration  ___/___ | | |
|  | Nasopharyngeal material collection  □Yes □No □Ignored | Month and year of collection  ___/___ | Culture result  □Positive □Negative  □Not done □Ignored | |

| Control Measures | Identified intimate contacts?  □Yes □No  □Ignored | If so, how many?  _________ | How many secondary cases have been confirmed among contacts?  □None □One  □Two or more □Ignored | |
| --- | --- | --- | --- | --- |
|  | Collected nasopharyngeal material from contacts?  □Yes □No  □Ignored | If so, how many?  _________ | How many contacts with a positive result?  _________ | Prevention and Control Measures  □Yes  1-Vaccination  2-Chemoprophylaxis  3-Both  □No □Ignored |

| Conclusion | Final Classification  □Confirmed □Discarded | Confirmation criteria  □Laboratory □Clinical epidemiological □Clinical |
| --- | --- | --- |
|  | Work-related disease  □Yes □No □Ignored | Evolution  □Cure □Death by pertussis □Death from other causes □Ignored |
